# Decreasing Frequency of Osteonecrosis of the Jaw in Cancer and Myeloma Patients Treated with Bisphosphonates: The Experience of the Oncology Network of Piedmont and Aosta Valley (North-Western Italy)

**DOI:** 10.1155/2013/672027

**Published:** 2013-02-27

**Authors:** Vittorio Fusco, Claudia Galassi, Alfredo Berruti, Cinzia Ortega, Libero Ciuffreda, Matteo Scoletta, Franco Goia, Mario Migliario, Anna Baraldi, Mario Boccadoro, Anastasios Loidoris, Oscar Bertetto

**Affiliations:** ^1^Medical Oncology Unit, Department of Oncology and Hematology, Ospedale SS Antonio e Biagio e C Arrigo, Via Venezia 16, 15121 Alessandria, Italy; ^2^Unit of Cancer Epidemiology, S. Giovanni Battista-Molinette and CPO-Corso Bramante 88, 10126 Piedmont, Turin, Italy; ^3^Oncology Unit, University of Brescia, Spedali Civili Hospital, Piazzale Spedali Civili 1, 25123 Brescia, Italy; ^4^Oncology Unit, IRCC Candiolo, Strada Provinciale 142, 10060 Candiolo, Turin, Italy; ^5^Oncology Unit, S. Giovanni Battista, Molinette, Corso Bramante 88, 10126 Turin, Italy; ^6^Oral Surgery Unit, S. Giovanni Battista, Molinette, Corso Bramante 88, 10126 Turin, Italy; ^7^Oral Care Unit, Mauriziano Hospital, Via Magellano 1, 10126 Turin, Italy; ^8^Dental Clinic, Department of Health Science, University of Eastern Piedmont “A. Avogadro”, Via Solaroli 17, 28100 Novara, Italy; ^9^Hematology Unit, Department of Oncology and Hematology, Ospedale SS Antonio e Biagio e Cesare Arrigo Hospital, Via Venezia 16, 15121 Alessandria, Italy; ^10^Hematology Unit, Turin University, Corso Bramante 88, 10126 Turin, Italy; ^11^“Rete Oncologica di Piemonte e Valle d'Aosta” Oncology Network, Azienda Ospedaliero Universitaria San Giovanni Battista-Presidio San Lazzaro, Via Cherasco 23, 10126 Turin, Italy

## Abstract

*Background*. Data concerning frequency of Osteonecrosis of Jaws (ONJ) are mostly based on single center experiences. *Patients and Methods*. Since 2005 a multidisciplinary study group collected data of cases of ONJ in patients treated with Bisphosphonates (BP) and observed in oncology and hematology centers of a regional network. *Results*. By December 2008, 221 cases were registered. We report details of 200 cases, identified after cross-checking reports from centres of medical oncology, haematology, and oral care. Primary neoplasm was breast cancer (39%), myeloma (32%), prostate cancer (16%), and other types of cancer (8%). In about 50% of the cases a history of dental extraction was present. Zoledronic acid was administered (alone or with other BP) to 178 patients (89%). Median time from first infusion to ONJ diagnosis was 21.0 (zoledronic acid only) and 39.0 months (pamidronate only). The number of ONJ cases per year was 3 in 2003, 21 in 2004, 58 in 2005, 60 in 2006, 37 in 2007, and 21 in 2008. *Conclusion*. The number of new ONJ cases in cancer and myeloma patients increased until 2006 and then reduced. The possible reasons of this trend (introduction of zoledronic acid; increase of ONJ awareness; diffusion of preventive dental measures; late modifications of BP prescription) are herein discussed.

## 1. Introduction

Bisphosphonates (BPs) are widely prescribed at different doses, way of administration (oral versus IV) and treatment durations, for a range of bone diseases including cancer-induced bone disease, osteoporosis, and Paget's disease. Most of IV prescriptions of more potent BPs are written for patients affected by myeloma or bone metastases from solid cancer [1]. Despite the optimal duration of treatment with BPs is unknown, several recommendations and position papers suggested in the past that administration of BP should be carried on indefinitely [2–4].

Osteonecrosis of the Jaws (ONJ) was first reported in patients treated with BPs in North American literature in 2003 [5, 6]. Ever since a large amount of case reports and case series have been published worldwide [6, 7], although, quite surprisingly, no cases were reported in previously published randomized controlled trials (RCT) that led to the introduction of BP in the therapy armamentarium.

The paucity of data from well-designed prospective trials do not allow to estimate the precise incidence of ONJ among subjects exposed to BPs [8–11]. According to several published reports (cohort studies, case series, usually based on single centre experience), the frequency of ONJ in cancer patients receiving BPs is estimated to vary in a wide range (i.e. from less than 1% to about 50%), suggesting that it can be a common event [12–14]. There are very few data about frequency of ONJ based on systematically collected data in large populations [6, 15].

The risk of ONJ is directly related to the cumulative dose (dose *per* duration of exposure) and the potency of BPs, with evidence of a greater risk with IV amino-BPs, and in particular with zoledronic acid [12, 16–19]. The role of other possible risk factors is at present unclear [20]; there are suggestions of an increased risk of ONJ in subjects exposed to antiangiogenic drugs as well [21, 22]. In most of cases, the trigger events leading to ONJ diagnosis are dental extraction, bone surgery, periodontal disease, and denture trauma, even though spontaneous cases are reported in almost all the published case series [16, 17, 23].

Measures to minimize the risk of ONJ have been proposed [12]; however, most of the advice is empirically based [20]. The utility of a population-based prospective registry has been claimed [20, 24].

We present here the results of a large series of ONJ cases systematically collected at oncology and hematology units and at referral oral care centers of a regional area in North-Western Italy: Piemonte and Valle d'Aosta.

## 2. Materials and Methods

In November 2005, a regional ONJ Study Group was established by members of a Piemonte and Valle d'Aosta network (Rete Oncologica), encompassing all public oncology and hematology units of two administrative regions in North-Western Italy. The group included maxillofacial surgeons and dental specialists of all referral centers in the regional area.

A data collection form was sent to all oncology and hematology units of the network and to the referral oral care centers (maxillofacial surgery; otolaryngology; hospital oral medicine units) in the region.

We cross-checked data from network centers and specialist units (to integrate data and to avoid double-counting), identifying cases observed before November 2005 with sufficiently detailed forms to be included in a specific data base [25].

From November 2005 onwards, data about new suspected and recognized ONJ cases were collected prospectively and sent (in case of confirmed ONJ diagnosis) to the study group coordinator (V.Fusco).

One author (A.Loidoris) visited most of the oncology and hematology units and maxillofacial or oral care units, to assure consistency in data collection. Local audit visits have been periodically repeated in the course of subsequent years in the main referral centers.

Cases were confirmed and included in the data base as “ONJ cases” when the following criteria were met: history of BP therapy; intraoral lesion (exposed bone, pus exudates, or fistulae) or extra oral manifestations of swelling or fistulae that fail to heal within 8 weeks; imaging exams identifying jaw bone necrosis in case of absent bone exposure; and no history of radiation therapy that could imply osteo-radionecrosis.

Requested and collected data included the following:

- demographics: age, sex;
- cancer and myeloma history: type of primary cancer or myeloma; date of diagnosis (myeloma or primary solid tumor, and metastatic bone lesions);
- BP therapy: start date, treatment duration, type of BP, dosage, and number of administrations;
- other cancer and myeloma treatments: chemotherapy (administered prior, concomitantly or after BP therapy), hormone therapy, steroid treatment (massive or occasional), antiangiogenic therapies;
- possible ONJ risk factors: smoking history, diabetes;
- site(s) (maxillary versus mandible) and date of ONJ diagnosis;
- clinical findings at the ONJ diagnosis;
- dental comorbidities or possible precipitating events, such as teeth extraction, periodontal surgery, dental implants, or traumatic use of dentures;
- ONJ treatment and evolution.

Continuous data are presented as median (interquartile range, IQR) or mean (Standard Deviation, SD) as appropriate, and categoric data as number (percentage, %). STATA SE version 11.0 (STATA Corp, USA) was used for all analyses.

## 3. Results

### 3.1. Patients Characteristics

We collected individual data of 241 patients identified as ONJ cases by the medical or dental specialists. Twenty patients had ONJ diagnosis after BP therapy of bone disease (osteoporosis and osteopenia, Paget's disease, etc.) without any clear history of cancer and were therefore beyond the subject of this paper. Twenty-one ONJ cases were excluded due to incomplete data regarding information of interest, that is, cancer history, BP treatment (duration, dose), and dental history.

The characteristics of the 200 ONJ cases included in this study are presented in Table 1.

The median time from diagnosis of the bone metastasis or myeloma to ONJ onset was 34.1 (range 3.5–268.4) and 42.7 months (5.6–203.2), respectively.

### 3.2. Bisphosphonates: Drug, Duration of Treatment, and Cumulative Dosage

One hundred forty-seven patients (73.5%) were treated with only one type of BP, 53 patients (26.5%) with at least two different drugs, being zoledronic acid the most prescribed drug (90% of total population) (Table 1).

The median time from the first infusion of BP to the diagnosis of ONJ was 27.4 months (range 1.8–203.1), with 20.7 months (range 1.8–74.5) for those receiving zoledronic acid only and 38.6 months (range 5.5–102.7) for those treated with pamidronate only. Among patients who received more than one BP, the median time was 56 months (range 6.5–203.1) with pamidronate/zoledronate sequence. The median duration of BP therapy and median doses of administered drugs are illustrated in Table 2.

### 3.3. Exposure to Possible Risk Factors and Medical Comorbidities

Among ONJ patients (Table 3), 17.5% were active smokers and 7.5% were ex smokers. About 45% of patients received massive steroids treatment (mostly in myeloma affected patients) and another 20% of patients were occasionally treated with steroids (mainly in association with chemotherapy). Diabetes was reported in 14% of the cases. About medical treatment timing, 15% had been treated with chemotherapy before the infusions of BPs only, 42% received chemotherapy concomitant to BP treatment, 17.5% had both prior and concomitant chemotherapy and 18.5% did not receive chemotherapy. Thirty-eight patients received anti-angiogenic drugs (35 thalidomide, 2 lenalidomide, and 1 bevacizumab). In about 50% of cases a history of dental extraction was present.

### 3.4. ONJ Clinical Manifestations

The presentation and treatment outcomes of ONJ are resumed in Table 3. The majority of patients (122) had a lesion in the mandible; in 57 patients, ONJ lesions occurred in the maxilla and in 21 patients in both maxilla and mandible. A considerable proportion of patients (48 cases, 24%) had multifocal lesions.

### 3.5. Followup and Treatment Outcome

The ONJ treatment was substantially uniform among centers. The therapeutic approach to each patient was changed on the basis of patient characteristics (age, Performance Status, comorbidities), disease extension (limited versus extended), expected cancer survival and prognosis.

Patients showing exposed and necrotic bone regions (but not heavily symptomatic) were generally followed and treated conservatively with local wound care and irrigations with/without antibiotics. Surgical procedures included conservative surgery (sequestrectomy or soft debridement), but in some selected cases mandibular resections and maxillectomies were performed.

After a short-term reevaluation (3–6 months after ONJ diagnosis and first treatments), a complete ONJ remission was observed in 11 patients (5.5%), whereas the disease improved in 69 patients (34.5%), did not change in 53 patients (26.5%), and worsened in 46 patients (23.0%). BP treatment was withdrawn in most patients (87.5%) at the ONJ occurrence.

### 3.6. Changes in Number of ONJ Cases over Time

**Figure 1:**
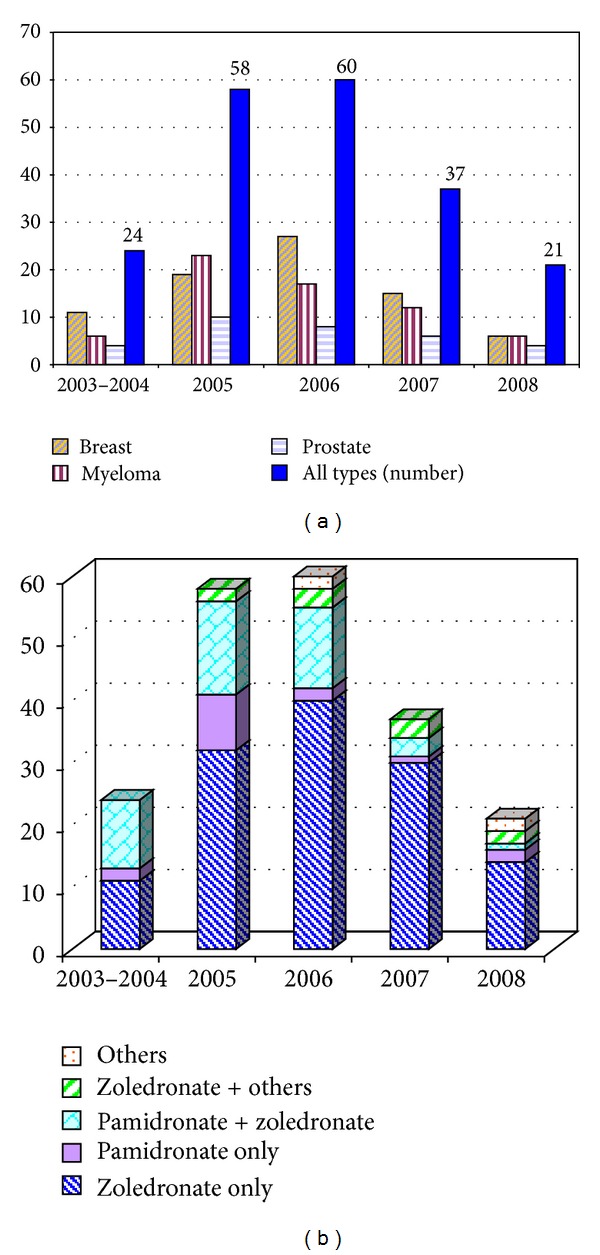
(a) Number of ONJ cases by type of disease and by year. (b) Number of ONJ cases per administered BP and by year.

The pattern of distribution of ONJ cases over time is depicted in Figure 1. It showed an initial increase from 2003 to 2006 and a subsequent decrease in the following 2 years. In particular the number of new diagnoses decreased by 65% in 2008 (21 cases) as compared to 2006 (60 cases) (Figures 1(a) and 1(b)). There was no substantial difference among the main patient groups (breast cancer, myeloma, prostate cancer, and other types of carcinoma) (Figure 1(a)). The drug (or sequence of drugs) administered to patients is shown in Figure 1(b).

## 4. Discussion

Osteonecrosis of jaws (ONJ) is a relatively new disease, recognized since 2003, potentially affecting the quality of life of cancer and myeloma patients [26]; a largely accepted definition (with accompanying staging system) has been established only in 2007 [27] and revised in 2009 [12], but the presence of cases without bone exposure enlarged the recognition problem [28, 29]. Incidence of ONJ is possibly underestimated worldwide [6, 7, 30].

In the present study we describe a large case series of ONJ cases among cancer and myeloma patients in the period 2003–2008. ONJ cases observed in all referenced regional centers have been prospectively collected since 2005, so that we are confident that very few patients could have escaped our registration after this date. Unfortunately, due to the absence of a unique collecting data system for all the patients treated with BPs in our region, we were not able to draw the incidence of ONJ among the exposed (at risk) population, but we could only estimate the crude frequency in the general population. This represents a clear limitation of our study.

Our results show that this adverse event is not rare, since we recorded a total of 221 cases among patients with cancer observed within a 5-6 year time period in a region of 4.3 million population. These numbers appear much higher than those previously reported in Australia (114 new cases in 2 years in a population of 20.3 millions) [15]. To our knowledge, no further data or estimations on large populations have been published. Projecting these data on a national basis and assuming an equivalent use of BPs in cancer patients in all the Italian regions, we could estimate that between 2000 and 3000 new ONJ cases have been observed in Italy (58 millions) from 2003 to 2008. These numbers are much higher than those reported to the Drug Surveillance System of the Italian Medicine Agency (425 cases on June 2009) [31].

**Figure 2:**
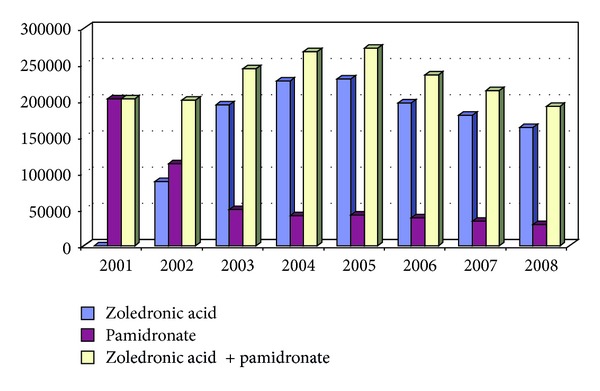
Number of pamidronate (1 unit = 90 mg) and zoledronate (1 unit = 4 mg) units prescribed in Italy between 2001 and 2008 (elaboration from AIFA data).

The number of new ONJ diagnoses sharply increased from 2003 to 2006, although active data collection in our region only started in November 2005, and therefore we cannot exclude that some ONJ cases diagnosed before this date could have been missed. From 2003 onwards an increasing number of ONJ reports appeared in dental and oncology literature leading to an enlarging awareness of the disease and preventing new ONJ cases from being misclassified as bone jaw metastasis, myeloma disease sites, osteomyelitis, and so forth, as occurred before [5, 6]. In addition, due to a possible “harvesting” effect, some prevalent cases could have been included as “new” cases in the first years. The increasing number of ONJ cases in our region parallels an increased use of zoledronic acid. In Italy, zoledronic acid, the most potent BP, rapidly and largely replaced pamidronate from 2002 onwards as the first choice BP therapy in myeloma and cancer patients (Figure 2), and many patients already receiving pamidronate were shifted to zoledronic acid. A trend comparable to the one observed in Italy was registered in the Piedmont region for the prescriptions of zoledronic acid.

The decreasing number of ONJ cases observed from 2006 onwards can have several explanations, but two factors appear as the most relevant. Firstly, the awareness of this important adverse event led from late 2005 onwards to the adoption in our region of preventive dental measures including a pretherapy dental assessment (with eventual necessary dental extractions before starting BP treatment, when possible) and limitation of further dental and jaw bone trauma during BP treatment; furthermore, periodical dental visits were recommended, and the patients were alerted to refer to a physician in case of dental problems arising during BP treatment. Secondly, a significant reduction in IV BP prescriptions has been observed in Italy (Figure 2), mainly due to a reduction in the zoledronic acid prescriptions, and there was a similar trend in the Piedmont region. This could be related to a lower number of treated patients or to a lower number of administered doses per single patient (a possible reduction of the individual cumulative dose due, for instance, to a reduced duration of treatment, as suggested by several authors and expert panels since 2006) [32–37], or both.

Both the above-mentioned factors (i.e., implementation of dental preventive measures and reduction in zoledronic acid prescriptions) could have contributed to the decreasing incidence trend of ONJ observed after 2006. However, the design of the present study does not allow to quantify the relative effects of these two factors in the reduction of the ONJ cases.

Two papers firstly reported on the efficacy of preventive dental measures [38, 39]. Both papers showed a reduction (but not the disappearance) of ONJ new cases in the time period in which these measures were adopted as opposed to an earlier period in which this was not done. These papers, however, did not adequately evaluate the contribution of a possible reduction of BP treatment duration and/or BP cumulative dose. Further studies with an appropriate prospective design are necessary to disentangle this point. Moreover, prospective randomized clinical trials (RCTs) are urgently needed to evaluate the risk/benefit ratio of alternative dosing schedules, as already suggested [20].

The physiopathology of ONJ is mostly unknown, but several predisposing factors have been pointed out, such as previous or concomitant treatment with steroids (massive prescriptions, as in myeloma patients) and antiangiogenic drugs. In our series these therapies had been administered in 45% and 19% of patients, respectively. The main apparent “trigger” condition leading to ONJ occurrence or discovery was dental extraction (50% of cases in our series), confirming the literature findings [14, 40, 41]. It has been stated that ONJ often occurs in older medically compromised patients [42]. Our data do not confirm this observation since the mean age of our ONJ patients (68 years) is not different from the expected age of cancer patients.

Most cases (89%) in our series had been treated with zoledronic acid, either alone or after a previous pamidronate therapy. In addition, a shorter latency between the BP start and the ONJ onset has been observed in zoledronic acid treated patients (median: 21 months) as opposed to pamidronate treated patients (median: 39 months). All these findings are confirmatory of literature data [19, 43]. In our regional experience, about 35% of the cases had their ONJ condition improved and in a further 5% the disease achieved a complete remission after a short followup. These data suggest that the conservative therapeutic approaches commonly employed seem efficacious in temporarily controlling the disease in some cases, but better evaluations of surgical treatments are needed, especially monitoring both mucosal and bone defect restoration upon a longer followup.

In conclusion, in this series we confirm that BP-associated ONJ was not a rare event in our region, even though its frequency has sharply decreased after 2006. This appears related to a greater awareness of this relevant adverse event and consequently to the adoption of several measures that include preventive and periodical dental visits, patient's education, and a reduction of BP cumulative dose. Further evaluations on years 2009–2011 in our regional network are ongoing.

## Figures and Tables

**Table 1:** Characteristics of ONJ patients.

	*N*	%
Total	200	
Age	Mean 68 years (SD: 9.23 years)	
Gender		
Female	121	60.5
Male	79	39.5
Bone disease		
Metastatic breast cancer	78	39.0
Myeloma	64	32.0
Metastatic prostate cancer	32	16.0
Bone metastases of other types of cancer*	16*	8.0
Osteoporosis in cancer patients	10	5.0
Bisphosphonates		
Zoledronate iv only	127	63.5
Pamidronate iv only	16	8.0
Pamidronate iv/zoledronate iv	43	21.5
Alendronate os	3	1.5
Clodronate im	1	0.5
Zoledronate iv/pamidronate iv	5	2.5
Zoledronate iv/ibandronate iv	3	1.5
Zoledronate iv/ibandronate os	1	0.5
Alendronate os/pamidronate iv/zoledronate iv	1	0.5

*Lung n.9; kidney n.3; bladder n.2; rectum n.1; occult primary n.1.

**Table 2:** Duration and dose of bisphosphonate treatment at the ONJ onset time.

Drug	No.	Median duration and range (months)	Median dose and range (mg)
Zoledronate only	127	20.7 (1.8–74.5)	72 (12–212)
Pamidronate only	16	38.6 (5.5–102.7)	2085 (360–4080)
Pamidronate/zoledronate	43	56.0 (6.5–203.1)	PAM 1080 (90–4680)/ZOL 88 (16–216)
Zoledronate/pamidronate	5	28.3 (10.8–38.3)	ZOL 48 (40–108)/PAM 270 (90–1260)

**Table 3:** Clinical characteristics and treatment outcomes of ONJ cases.

	*N*	%
*Bone involved *		
Mandible only	122	61.0
Maxilla only	57	28.5
Maxilla and mandible	21	10.5
*Possible risk factors *		
Smoking habit		
Active smokers	35	17.5
Past smokers	15	7.5
Never smokers	146	73.0
Unknown/unavailable	4	2.0
Steroids		
None	64	32.0
Massive	91	45.5
Occasionally	40	20.0
Unknown	5	2.5
Diabetes mellitus	28	14.0
Chemotherapy		
None	35	17.5
Prior	32	16.0
Concomitant	84	42.0
Prior and concomitant	35	17.5
Unknown	14	7.0
Endocrine therapy	98	49.0
Antiangiogenic drugs	38	19.0
*Dental history *		
Extraction	94	47.0
Denture	20	10.0
Parodontopathy	7	3.5
Implant	2	1.0
No risk factors reported	62	31.0
Unknown/unavailable	15	7.5
*Treatment of ONJ *		
Nonsurgical	79	39.5
Curettage only	92	46.0
Major surgical	15	7.5
Unknown/unavailable	14	7.0
*Outcome (3–6 months after ONJ diagnosis and first treatment) *		
Resolved	11	5.5
Improved, persistent	69	34.5
Stable, persistent	53	26.5
Worsened	46	23.0
Dead before evaluation	11	5.5
Unknown/unavailable	10	5.0
*Survival by December 2008 *		
Median followup (months)	13.8	IQR:18.4
Dead	72	36.0
